# Radiation-Induced Changes in Tumor Vessels and Microenvironment Contribute to Therapeutic Resistance in Glioblastoma

**DOI:** 10.3389/fonc.2019.01259

**Published:** 2019-11-15

**Authors:** Yun-Soo Seo, In Ok Ko, Hyejin Park, Ye Ji Jeong, Ji-Ae Park, Kwang Seok Kim, Myung-Jin Park, Hae-June Lee

**Affiliations:** ^1^Division of Radiation Biomedical Research, Korea Institute of Radiological & Medical Sciences, Naju, South Korea; ^2^Herbal Medicine Resources Research Center, Korea Institute of Oriental Medicine, Naju, South Korea; ^3^Division of Applied RI, Korea Institute of Radiological & Medical Science, Seoul, South Korea

**Keywords:** glioblastoma, glioma stem cell, radiotherapy, tumor microenvironment, tumor vessel

## Abstract

Glioblastoma (GBM) is a largely fatal and highly angiogenic malignancy with a median patient survival of just over 1 year with radiotherapy (RT). The effects of RT on GBM remain unclear, although increasing evidence suggests that RT-induced alterations in the brain microenvironment affect the recurrence and aggressiveness of GBM. Glioma stem cells (GSCs) in GBM are resistant to conventional therapies, including RT. This study aimed to investigate the effect of radiation on tumor growth and the GSC microenvironment in a mouse model of glioma. To evaluate the growth-inhibitory effects of ionizing radiation on GSCs, tumor volume was measured via anatomical magnetic resonance imaging (MRI) after the intracranial injection of 1 × 10^4^ human patient-derived GSCs (83NS cells), which exhibit marked radioresistance. When a tumor mass of ~5 mm^3^ was detected in each animal, 10 Gy of cranial irradiation was administered. Tumor progression was observed in the orthotopic xenografted GSC tumor (primary tumor) from a detectable tumor mass (5 mm^3^) to a lethal tumor mass (78 mm^3^) in ~7 d in the non-irradiated group. In the RT group, tumor growth was halted for almost 2 weeks after administering 10 Gy cranial irradiation, with tumor growth resuming thereafter and eventually approaching a lethal mass (56 mm^3^) 21 d after radiation. Radiation therapy yielded good therapeutic effects, with a 2-fold increase in GSC glioma survival; however, tumor relapse after RT resulted in higher mortality for the mice with a smaller tumor volume (*p* = 0.029) than the non-irradiated tumor-bearing mice. Moreover, tumor regrowth after IR resulted in different phenotypes associated with glioma aggressiveness compared with the non-irradiated mice; the apparent diffusion coefficient by diffusion MRI decreased significantly (*p* < 0.05, 0 Gy vs. 10 Gy) alongside decreased angiogenesis, abnormal vascular dilatation, and upregulated CD34, VWF, AQP1, and AQP4 expression in the tumor. These findings demonstrate that radiation affects GSCs in GBM, potentially resulting in therapeutic resistance by changing the tumor microenvironment. Thus, the results of this study suggest potential therapeutic targets for overcoming the resistance of GBMs to RT.

## Introduction

Glioblastoma (GBM) is the most malignant and highly angiogenic tumor in the central nervous system (CNS). Due to its aggressiveness, GBM has a very poor prognosis, with a median survival of 14.6 months from diagnosis (1–3). Radiotherapy (RT) is currently used to treat GBM alongside surgical resection and chemotherapy. Highly proliferative cells, including tumor cells, are sensitive to ionizing radiation-induced DNA damage via reactive oxygen species, which causes apoptosis and reduces cell proliferation. Although RT is a locoregional treatment for GBM, RT-induced alterations in the brain microenvironment have been shown to contribute to GBM recurrence and aggressiveness (4). Understanding of the origin of therapeutic resistance in GBM may therefore help to improve patient outcome and survival.

A specific subpopulation of glioma cells, known as glioma stem cells (GSCs), contribute to tumor recurrence during aggressive multimodal therapies. After RT, GBM frequently recurs as focal masses (5), indicating that GSCs are radio-resistant and responsible for relapse (6, 7). GSCs can inherently resist conventional therapy due to their enhanced self-renewal and differentiation potential (8). Recent studies reporting that GSCs maintain GBM (9) have indicated that GSCs interact closely with the vascular niche and promote neovasculogenesis by releasing angiogenic factors (10); however, the underlying mechanisms remain unclear.

GBM patients have poor prognosis due to tumor cells that survive initial treatment and cause relapse or recurrence; thus, failure to inhibit tumor growth at the primary site is a major cause of mortality (11). GBM tumors originate in the brain and interact closely with their unique microenvironment (12). Furthermore, highly aggressive tumors rarely metastasize outside the brain (13), indicating a preference for the brain microenvironment. Cell-cell interactions, tissue dynamics, and cytokines and growth factors constitute a complex microenvironment that is altered in the presence of GBM tumors, promoting tumor invasion, and therapeutic resistance. Therefore, it is important to identify and characterize treatment-resistant tumor cells and whether they influence their microenvironment.

In this study, we investigated the extent of GSC glioma progression after brain irradiation for a specific duration via magnetic resonance imaging (MRI) with an orthotopic xenograft mouse model of GBM established using patient-derived GSCs. Furthermore, we assessed the morphological and molecular phenotypes that may be associated with radioresistance in post-irradiation relapse.

## Materials and Methods

### Cell Culture

The GBM patient-derived GSC line 83NS was maintained in DMEM/F-12 (Corning, 10-090-cvr, Corning, NY, USA) supplemented with B27 (Invitrogen, 17504044, Carlsbad, CA, USA), epidermal growth factor (10 ng/mL; Prospec, cyt-217, Brunswick, NJ, USA), and basic fibroblast growth factor (5 ng/mL; Prospec, cyt-218). 83NS cells were provided by Dr. Ichiro Nakano (University of Alabama at Birmingham, Birmingham, AL, USA) (14). Prior to transplantation, cells were washed with PBS and dissociated. After centrifugation at 1,500 rpm for 5 min, the cell pellet was resuspended in PBS at a density of 1 × 10^4^ cells/3 μL.

### Establishment of the Orthotopic Mouse Model of Glioma and Radiotherapy

All animal experiments were conducted in accordance with protocols approved by the Institutional Animal Care and Use Committee (IACUC) of the Korea Institute of Radiological and Medical Sciences (KIRAMS2018-0079). Athymic BALB/c nu/nu mice were purchased from Orient Bio Inc. (Seoul, Korea), housed under specific pathogen-free conditions, and supplied with standard rodent feed and tap water *ad libitum*. The animals' heads were fixed in a stereotactic frame using non-perforating bars, a midline incision was made on the scalp, and a burr hole was drilled 0.1 mm posterior and 2 mm left of the bregma. An 83NS cell suspension (1 × 10^4^ cells) was injected into the left frontal cortex at the coordinate bregma using a stereotactic frame and microinjector. The average tumor size was determined to be 5 mm^3^ by MRI. Mice were randomly divided into two subgroups: a control group and an irradiated group. Radiation (a single dose of 10 Gy) was administered to the entire head under anesthesia (30 mg/kg Zoletil and 10 mg/kg Rompun) using an X-Rad320 (Precision X-Ray, East Haven, CT, USA; filter: 2 mm AI; distance: 42 cm; 260 kVp, 10 mA, 10Gy/5 min).

### MRI

All MRI was performed using a 9.4-T animal MR system and a specific mouse brain coil (Agilent Technologies, Palo Alto, CA, USA). The incidence and size of the orthotopic glioma were determined radiologically. Cranial irradiation was carried out 12 d after stereotactic 83NS cell transplantation when the tumor volume approached 5 mm^3^. To confirm the therapeutic effects of tumor irradiation, MR images were obtained 0, 3, 7, 14, and 21 d after irradiation and tumor size was compared. Before MRI, animals were anesthetized with 2.5% isoflurane in oxygen. A fast spin-echo MR sequence for T2-weighted imaging (T2-WI) was used with the following parameters: repetition time (TR), 2500 ms; effective echo time (TE_eff_), 30 ms; echo train length (ETL), 4; average number, 4; slice thickness, 0.8 mm; slice number, 6; matrix size, 192 × 192; field-of-view, 25 × 25 mm^2^; and total imaging time, 605 s. Tumor volume was measured by summing all voxels within the tumor boundary of the anatomical T2-W images using ImageJ software (NIH Bethesda, MD, USA).

A fast spin-echo MR sequence for diffusion-weighted imaging was used with the following parameters: repetition time (TR), 2000 ms; echo time (TE), 23.5 ms; echo number, 1; average number, 1; slice thickness, 0.8 mm; matrix size, 192 × 192; field-of-view, 25 × 25 mm^2^; b-value, 0 and 800 s/mm^2^; and total imaging time, 512 s. Apparent diffusion coefficient (ADC) maps and values were processed using the intrinsic VnmrJ 4.0 workstation (Agilent Technologies, Inc.).

### Histopathological Analysis

All mice were euthanized by CO_2_ inhalation and brain tissue specimens were harvested in accordance with IACUC guidelines. The harvested brain tissue specimens were fixed in 4% paraformaldehyde, embedded in paraffin, and cut into 5 μm sections using a microtome (Leica, Nussloch, Germany).

Immunofluorescence staining was performed as described previously (15). Briefly, specimens were blocked with blocking buffer (PBS with 1.5% normal horse serum and 0.1% Triton X-100), incubated with anti-Ki-67 (Acris, DRM004, Herford, Germany), anti-CD31 (R&D Systems, AF3628, Minneapolis, MN, USA), anti-CD34 (SantaCruz, sc-74499, Dallas, TX, USA), anti-Von Willebrand factor (VWF; Abcam, ab6994, Cambridge, MA, USA), anti-aquanporin 1 (AQP1; SantaCruz, sc-32737), and anti-aquaporin 4 (AQP4; Novus Biologicals, NBP1-87679, Littleton, CO, USA) antibodies overnight at 4°C, and then incubated with Alexa Fluor 488-, Alexa Fluor 546-, and Alexa Fluor 647-conjugated secondary antibodies (Invitrogen). After washing the sections with PBST, nuclei were counterstained with DAPI and fluorescence visualized was using confocal microscopy (Carl Ziess, Oberkochen, Germany). Fluorescence intensity was measured using ImageJ software (NIH). Briefly, fluorescence positive areas were assessed and the ratio was calculated (16). A TUNEL assay was carried out to evaluate apoptotic glioma cells using an Apoptosis kit (Promega, g3250, Madison, WI, USA) in accordance with the manufacturer's instructions.

### Quantitative Reverse Transcription Polymerase Chain Reaction (qRT-PCR) Analysis

Total RNA was isolated from cultured GSCs treated with or without radiation (10 Gy) using an RNeasy Mini kit (Qiagen, Valencia, CA, USA) and 1 μg was reverse-transcribed into cDNA using amfiRivert cDNA Synthesis Platinum Master Mix (GenDEPOT, Barker, TX, USA) in accordance with the manufacturer's protocol. qPCR was carried out in triplicate, using qPCR SYBR green 2× mastermix kit (M Biotech, 18303, Seoul, Korea) with a CXF-96 detection system (Bio-Rad Laboratories, Hercules, CA, USA). Relative gene expression was normalized to that of *Gapdh* using the comparative C_T_ method with Bio-Rad CFX manager v2.1 (Bio-Rad Laboratories). The following primers were used: *aqp1* (sense: 5′-CGTGACCTTGGTGGCTCAG-3′; anti-sense: 5′-GGACCGAGCAGGGTTAATCC-3′), *aqp4* (sense: 5′-AACGGACTGATGTCACTGGC-3′; anti-sense: 5′-AAAGGATCGGGCGGGATTC-3′), and *Gapdh* (sense: 5′- CATCGCTCAGACACCATG 3′; anti-sense: 5′-TGTAGTTGAGGTCAATGAAGGG-3′).

### Statistical Analysis

Data are presented as the mean ± standard deviation (SD). Differences between tumor stages were assessed by one-way ANOVA with statistical significance set at *p* < 0.05. All statistical analyses were carried out using GraphPad Prism Software version 7.0 (GraphPad Software, La Jolla, CA, USA).

## Results

### Effect of IR on the Survival of Orthotopic GBM Xenograft Model Mice

**Figure 1:**
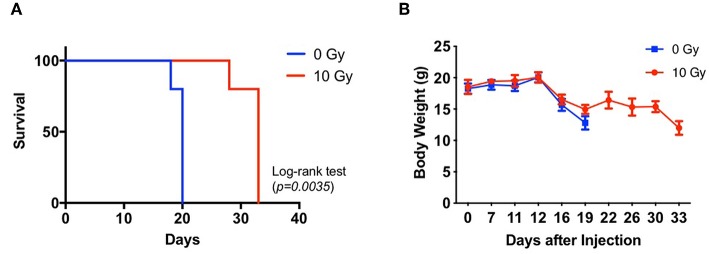
Effects of irradiation (IR) on the tumorigenicity of glioma stem cells (GSCs) in an orthotopic xenograft mouse model of glioma. **(A)** Kaplan-Meier survival graph of mice implanted with 83NS cells and treated with or without radiation (*n* = 5, 1 × 10^4^ cells injected per mouse). IR treatment was administered when tumors grew to their detectable size (12 d, 10 Gy). **(B)** Changes in body weight were monitored.

We assessed the survival of non-IR control and IR mice administered with 83NS cells. GBM xenograft model mice were orthotopically injected with 83NS cells and subjected to whole-brain RT (single 10 Gy dose) 12 d post-injection. As shown in Figure 1A, the IR group displayed significantly higher survival than the control group (*p* = 0.0035, Log-rank test) and the median survival of the IR group (32 ± 2.24) was 12.4 d longer than that of the control group (19.6 ± 0.89). We also monitored the body weight of each mouse. In the control group, body weight was maintained for 12 d after injection and then rapidly decreased when the tumors became detectable by MRI (Figure 1B). Weight loss in the IR group was temporarily delayed after IR but resumed 2 weeks later, achieving similar levels to the control group.

### IR Delayed Orthotopic GSC Glioma Progression

**Figure 2:**
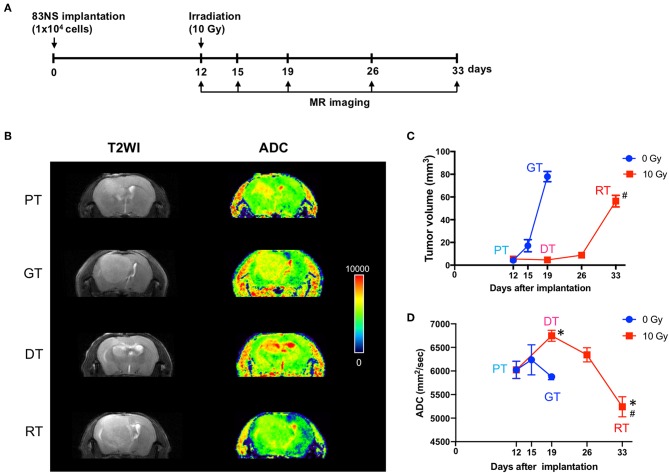
Effects of irradiation (IR) on tumor progression in an orthotopic mouse model of glioma. **(A)** Experimental protocol for IR and magnetic resonance imaging (MRI). **(B)** Representative images of brain imaging by anatomical or diffusion-weighted MRI of an orthotopic xenograft tumor model. **(C)** Graphical representation of changes in glioma volume during tumor progression with or without IR. The volume of each GSC glioma was measured using ImageJ software from anatomical MR images. **(D)** Apparent diffusion coefficient maps generated by diffusion-weighted MRI. Data presented as mean ± standard error of the mean. *p < 0.05. Significant differences among the tumor stages compared to PT were determined by one-way ANOVA (*n* = 4–6). ^#^*p* < 0.05. Significant differences between the GT and RT groups were determined using unpaired *t*-tests (*n* = 4–6). PT; primary tumors detectable by MRI; GT; growing tumors that progressed without IR; DT; delayed tumors after IR; RT; re-grown tumors after IR.

MRI has been widely used to characterize brain tumor growth, progression, and response to various treatments in clinical and preclinical studies (17, 18). To evaluate the effect of IR on GSC glioma, we recorded whole tumor volume using anatomical MRI. As shown in Figure 2, the tumors in the control group progressed in 7 d from a detectable mass of ~5 mm^3^ (5.50 ± 0.94) to a lethal mass of 78 mm^3^ (77.95 ± 4.47). In the IR group, tumor growth was temporarily attenuated for 14 d after RT; however, it eventually reverted to a lethal mass of ~56 mm^3^ (56.35 ± 5.12) after a further 7 d. Thereafter, we performed diffusion-weighted MRI (DW-MRI), which quantifies the movement of water within tumors by measuring the ADC, which is negatively correlated with tumor cell density. An increase in ADC values is a quantifiable indicator of antitumor efficacy and thus decreasing angiogenic activity and increasing apoptotic rate (17–19). Figure 2D shows that IR increased the ADC by up to 12% during the delay in GSC glioma growth (*p* < 0.05, one-way ANOVA, PT vs. DT), whereas the ADC remained largely unchanged during tumor progression in the non-IR group (+1% at 3 d and −3% at 7 d vs. 0 d). Based on GSC glioma relapse, the ADC was reduced by up to 13% 21 d after IR and was significantly lower than that of the non-irradiated group (*p* = 0.044, unpaired *t*-test, GT vs. RT), although the tumor volume of the relapsed tumor was significantly lower than that of the non-IR group (*p* = 0.029, unpaired *t*-test). Based on these results, increased ADC, which was induced by a decline in cell density and enlarged extracellular space after radiation, was decreased according to tumor relapse to an even greater extent than that in growing tumors (GTs), which suggests that RTs had a higher cell density than GTs. To evaluate the changes induced by IR and to allow additional analysis, the tumors were divided into four groups: primary tumors (PTs) that were detectable by MRI, growing tumors (GTs) that progressed without IR, delayed tumors after IR (DTs), and re-grown tumors after IR (RTs).

### IR Temporally Inhibited GSC Proliferation and Attenuated GSC Proliferation During Tumor Progression

**Figure 3:**
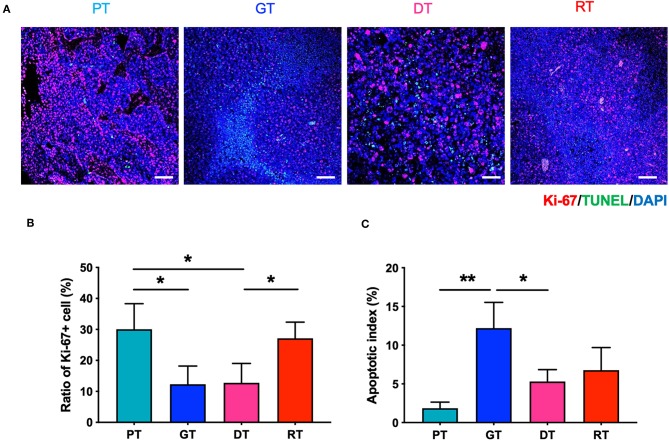
Effects of irradiation (IR) on the proliferation and apoptosis of tumor cells in the orthotopic mouse model of glioma. **(A)** Glioma from the brain tissue of xenografted mice were stained with hematoxylin and eosin to identify tumor regions. **(B)** Proliferative and apoptotic cells were visualized by immunofluorescence staining and TUNEL assays, respectively. Scale bar, 100 μm. **(C)** The percentage of Ki-67+ and TUNEL+ cells of total tumor cells per unit area was determined. *p < 0.05 and **p < 0.01. Significant differences among the treated groups were determined by analysis of variance followed by Tukey's test for multiple comparisons (*n* = 4–6). PT; primary tumors detectable by MRI, GT; growing tumors that progressed without IR, DT; delayed tumors after IR, RT; re-grown tumors after IR.

To investigate the effect of IR on GSC glioma growth, we enumerated the proliferative and apoptotic cells in the brain tumors. As shown in Figure 3, the number of Ki-67-positive proliferative cells decreased gradually in accordance with tumor growth in the GTs compared to the PTs, whilst the number of TUNEL-positive apoptotic cells increased. These results may have occurred due to spatial constraints during the progression of solid tumors which induce cell death and affect the delivery of oxygen and nutrients to the tumor (20). The number of Ki-67-positive cells decreased significantly 7 d after IR in the DTs compared to that in the PTs (*p* < 0.05), corresponding to the delay in tumor growth. These inhibitory effects on tumor growth are consistent with the low tumor cell density representing increased ADC in the DTs (Figures 2B,D). The number of Ki-67-positive cells was also significantly increased in RTs compared to that in DTs (*p* < 0.05) during tumor regrowth. Furthermore, no significant differences were observed in the apoptotic index during regrowth from DTs to RTs, unlike the tumor growth from PTs to GTs. Delayed tumor regrowth was accompanied by GSC re-proliferation and a significant decrease in ADC levels compared with GTs, indicating that 10 Gy of IR transiently inhibited GSC growth; however, GSCs regrew with increased aggressiveness.

### IR Suppressed Angiogenesis and Structurally Altered Microvasculature in the Late Phase of Tumor Progression

**Figure 4:**
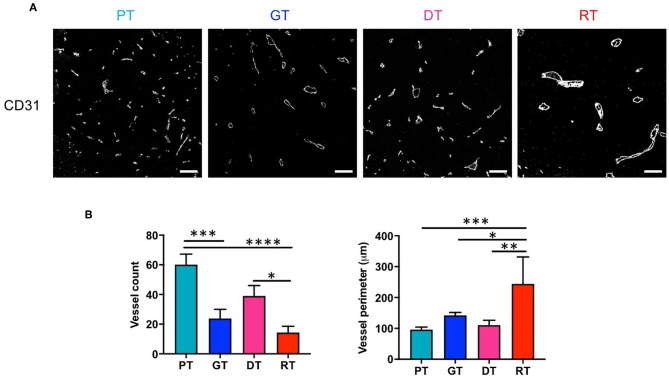
Effects of irradiation on microvessel composition in an orthotopic mouse model of glioma. **(A)** Microvessels in tumor tissues were visualized by immunofluorescence staining using an endothelial-specific anti-CD31 antibody. Scale bar, 100 μm. **(B)** The number and diameter of microvessels were determined. *p < 0.05, **p < 0.01, ***p < 0.005, and ****p < 0.0001. Significant differences among the treated groups were determined by analysis of variance followed by Tukey's test for multiple comparisons (*n* = 6). PT; primary tumors detectable by MRI, GT; growing tumors that progressed without IR, DT; delayed tumors after IR, RT; re-grown tumors after IR.

We investigated the effect of RT on GSC glioma angiogenesis by measuring the microvessel density (MVD) of tumors using CD31 as a pan-endothelial cell marker. As shown in Figure 4, MVD decreased significantly with tumor progression (*p* < 0.01, PT vs. GT) or re-growth (*p* < 0.05, DT vs. RT). During the early phase of tumor progression, IR slightly but non-significantly inhibited angiogenesis (*p* = 0.074, PT vs. DT), whereas in the late phase of tumor progression IR drastically inhibited MVD in RTs compared with PTs (*p* < 0.001) or DTs (*p* < 0.05). Vessel diameter increased sharply during delayed tumor regrowth compared to ordinary tumor progression from PTs to GTs, consistent with the decrease in ADC (Figure 2D). Interestingly, RTs showed a significant increase in vessel diameter compared to that in GTs, which had similar tumor sizes. These results indicate that radiation inhibits angiogenesis and alters the vasculature of GSC gliomas.

### IR Altered Vascular Phenotypes in GSC Glioma

**Figure 5:**
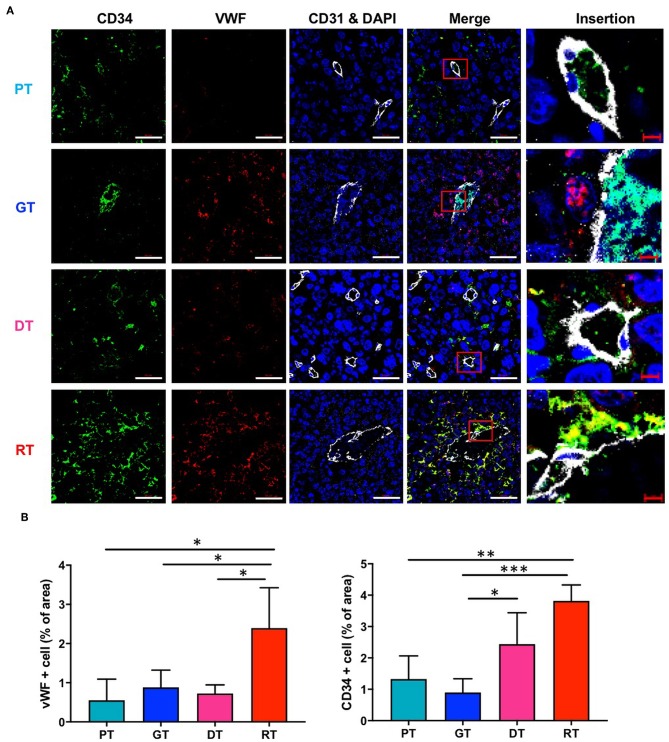
Effects of irradiation on the vascular microenvironment in an orthotopic mouse model of glioma. **(A)** Vascular components in tumor tissue were visualized by immunofluorescence staining using anti-CD34 and anti-VWF antibodies. Scale bar (white), 50 μm. Scale bar (red), 10 μm. **(B)** Proportions of CD34+ and VWF+ cells in tumor tissues were determined using ImageJ software. *p < 0.05, **p < 0.01, and ***p < 0.005. Significant differences among the treated groups were determined by analysis of variance followed by Tukey's test for multiple comparisons (*n* = 6). PT; primary tumors detectable by MRI, GT; growing tumors that progressed without IR, DT; delayed tumors after IR, RT; re-grown tumors after IR.

To verify the IR-induced morphological microvascular changes, we examined the molecular phenotypes of the tumor vessels by immunofluorescence staining with the additional endothelial cell markers CD34 and VWF during each stage of tumor progression. CD34 is a well-known marker of angiogenesis and endothelial progenitor cells (21, 22), whilst VWF is a multimeric plasma glycoprotein that mediates platelet adhesion to both the subendothelial matrix and endothelial surfaces (23) and is associated with tumor survival and angiogenesis (24, 25). As shown in Figure 5, in the non-irradiated group, CD34 and VWF expression were not markedly different in GTs compared to that in PTs. DTs showed only a mild increase in the number of CD34-positive cells and no changes in VWF expression in comparison with PTs, which displayed a similar tumor size. However, IR significantly increased the number of VWF- and CD34-positive cells in GTs compared to RTs. Due to regrowth, the tumors displayed decreased angiogenesis and enlarged vasculature; hence, increased CD34 and VWF may be involved in vascular abnormality rather than angiogenesis.

**Figure 6:**
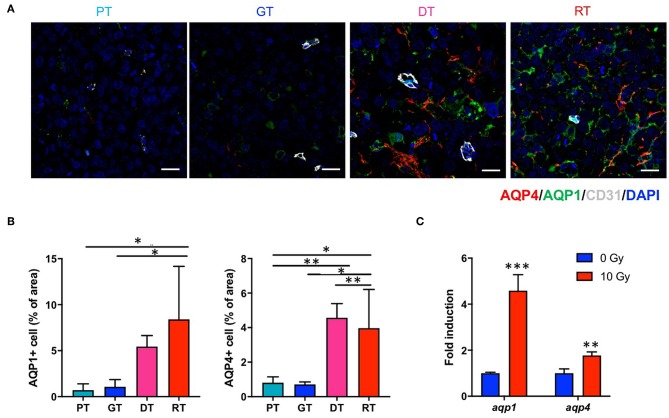
Effects of irradiation (IR) on the AQP expression in an orthotopic mouse model of glioma. **(A)** AQPs in tumor tissues were visualized by immunofluorescence staining using anti-AQP1 and anti-AQP4 antibodies. Scale bar, 100 μm. **(B)** The proportion of AQP1+ and AQP4+ cells in tumor tissues were determined using ImageJ software. *p < 0.05 and **p < 0.01. Significant differences among the treated groups were determined by analysis of variance followed by Tukey's test for multiple comparisons (*n* = 6). PT; primary tumors detectable by MRI, GT; growing tumors that progressed without IR, DT; delayed tumors after IR, RT; re-grown tumors after IR. **(C)** *aqp1* and *aqp4* mRNA expression levels were quantified by quantitative reverse transcription polymerase chain reaction analysis. 83NS cells were treated with or without IR (10 Gy) **p < 0.01 and ***p < 0.005. Significant differences between the 0 and 10 Gy groups were determined by unpaired *t*-tests (*n* = 3).

Since DW-MRI showed significant changes depending on the state of the tumor after IR, we also analyzed two major aquaporins (AQPs), AQP1 and AQP4, which are transmembrane water transporters that are primarily expressed in the brain tissue. In the non-irradiated group, AQP1 and AQP4 were expressed in GSCs at basal levels and no significant changes were observed in their expression during tumor progression. However, both AQPs were significantly upregulated following IR (*p* < 0.05; Figure 6). AQP1 is reportedly expressed in normal brain endothelial cells; however, AQP1 displayed a different expression pattern to CD31 (Figure 6A). Moreover, AQP4 is generally expressed in astrocytes; however, AQP4 displayed no colocalization with the astrocyte marker GFAP in the tumors (data not shown). We then examined *aqp1* and *aqp4* mRNA expression by qRT-PCR to determine whether IR upregulates AQPs in 83NS GSCs. As shown in Figure 6C, both AQPs were directly and significantly upregulated (*p* < 0.01) in 83NS 48 h after IR (10 Gy). These results show that IR directly alters GSCs and influences the microenvironment associated with tumor regrowth and aggressiveness.

## Discussion

GBM is one of the most intractable and angiogenic malignant tumors of the CNS. Despite recent advancements in the treatment of solid tumors, the treatment of these malignant gliomas remains essentially palliative since GBMs are extremely resistant to conventional radiation and chemotherapy. Moreover, despite significant technological improvements, radiotherapeutic effects are generally limited due to marked radioresistance in gliomas, particularly GSCs. Specific subpopulations of GSCs underlie this recurrence even when treated with aggressive multimodal therapies (26). In this study, we investigated the effects of therapeutic IR on GSCs in GBM by assessing histological and molecular alterations induced by IR in an orthotopic xenograft mouse model of GBM established using patient-derived GSCs. After IR, tumor progression was temporarily inhibited and the median survival increased for 12.4 days; however, tumors displayed rapid, and aggressive regrowth. Regrown GSC glioma, which displayed a smaller tumor volume than the non-irradiated group, was lethal to mice. Based on comparative histopathological analysis of different stages of tumor growth with or without IR, tumor regrowth after IR occurred alongside significant alterations in the vascular microenvironment. As shown in Figure 5, CD34 and VWF were significantly upregulated in regrown tumors with enlarged vessels. Unlike numerous studies reporting that CD34 and VWF are involved in angiogenesis, our study shows that CD34 and VWF expression coincide with reduced tumor vasculature. Consistent with our results, some studies have reported that primary GBM is characterized by increased angiogenesis, while recurrent GBM displays increased vasculogenesis and decreased angiogenic activity after RT (27). Hence, CD34 and VWF upregulation could be involved in abnormal vasculogenesis during tumor regrowth. Unlike ordinary vessel progression from PT to a GT which results from maturation and stabilization, vessel development from a DT to a RT after radiation may occur by vasculogenesis. Loss of angiogenic activity caused by radiation led to an influx of circulating cells which boost vasculogenesis, including endothelial progenitor cells and myeloid cells (3, 28). The increase in CD34+ and VWF+ cells in DTs and RTs caused by the infiltration of circulating cells might increase vessel diameter, which is crucial for tumor recurrence after radiation. Although CD34 is a marker of vascular endothelial progenitor cells (29, 30) and an optimal marker of microvascular density, a recent study showed that CD34 overexpression was associated with higher WHO glioma grades (III + IV) in 684 patients, suggesting that CD34 is a potential diagnostic and prognostic marker and therapeutic target for gliomas (31). In addition, VWF was only significantly upregulated in the late phase of GSC glioma regrowth; endothelial cell activation and the release of the procoagulatory protein VWF induce platelet aggregation, thus protecting cancer cells from immune cells, including NK cells, which is essential for malignancy (32). Therefore, these IR-induced alterations in the tumor microenvironment could contribute to resistance against further treatments, including RT.

This study demonstrated that IR upregulates AQP1 and AQP4 in GSC glioma tissue and 83NS cells *in vitro*. It has been reported that APQ expression is involved in water diffusion (33). To our knowledge, no studies have yet investigated IR-induced alterations in AQPs in GSCs or GBM. Since AQPs are involved in water transportation and edema, their upregulation in histopathological analysis was inconsistent with the reduced ADC in DW-MRI. In brain cancer, AQP1 expression is associated with brain capillary endothelial cells, which do not express AQP1 in normal brain tissue. The signals that induce AQP1 expression in the endothelium of brain tumors remain unclear but might include signals regulating the production and release of VEGF from cancer cells. As shown in Figure 6A, AQP1 was not expressed in tumor endothelial cells in GSC glioma. Hayashi et al. reported that AQP1 induction correlated with tumor cell metabolism and increased glycolysis and lactate dehydrogenase (LDH) activity, with patient GBM tissue exhibiting increased coincident AQP1, LDH, and cathepsin B expression levels which contributed to acidification of the extracellular milieu and glioma cell invasiveness (34). AQP4 is primarily an astroglial membrane protein localized in astrocytic endfeet that serves as a key functional component of the blood-brain barrier and is thought to be involved in brain edema pathogenesis (35). However, immunofluorescence staining and the ADC map of late phase regrown GSC glioma revealed differences compared to previous findings. In GSC glioma tissue, AQP4 was expressed on tumor cells, not tumor endothelial cells, and its expression levels decreased along with ADC levels, thus may not have contributed to edema. Interestingly, AQP4 expression is correlated with the incidence of epileptic seizures in GBM since patients with seizures have higher cell membrane AQP4 levels, suggesting that AQP4 expression is regulated post-transcriptionally (36). Furthermore, Lan et al. reported that AQP4 dissociates from orthogonal particle arrays and is redistributed across the entire surface of glioma cells under tumor conditions; thus, AQP4 expression levels may correlate with tumor grade as AQP4 expression increases in higher glioma grades (37). These previous studies support our finding that increased AQP expression after IR may be associated with GSC glioma malignancy or aggressiveness; however, further studies are required to elucidate the role and underlying mechanism of AQPs involvement in GBM radioresistance.

There are numerous obstacles to improving the therapeutic efficacy of RT and further studies are required to investigate the basic molecular events in GBM. In particular, GSCs are responsible for post-treatment GBM relapse. To understand the molecular events that occur in radioresistant tumors, we used an orthotopic mouse model of glioma implanted with a patient-derived GSC xenograft to monitor tumor progression after RT and alterations in the tumor microenvironment over time. This study shows that CD34, VWF, and AQPs are associated with post-IR GSCs glioma relapse and provides essential insights for the development of treatment regimens for radioresistant tumors.

## Data Availability Statement

All datasets generated for this study are included in the article.

## Ethics Statement

The animal study was reviewed and approved by the Institutional Animal Care and Use Committee at the Korea Institute of Radiological & Medical Sciences.

## Author Contributions

Y-SS: data collection, data analysis, and manuscript writing. IK, HP, and YJ: data collection and data analysis. J-AP: data analysis. KK and M-JP: developed the study concept project development. H-JL: project development, developed the study concept, edited, manuscript writing, and revised the manuscript.

### Conflict of Interest

The authors declare that the research was conducted in the absence of any commercial or financial relationships that could be construed as a potential conflict of interest.
